# Fire management effects on ruminal digestibility and in vitro methane emissions of subtropical rangeland plant species

**DOI:** 10.1093/tas/txad080

**Published:** 2023-07-18

**Authors:** Abmael S Cardoso, Maria L Silveira, Joao M B Vendramini, Philipe Moriel, Marta M Kohmann, Hiran M S Silva, Vinicius Izquierdo, Lais O Lima, Nauara M Lage Filho, Joao V L Silva, Joao M D Sanchez

**Affiliations:** Range Cattle Research and Education Center, University of Florida, Ona, FL 33865, USA; Range Cattle Research and Education Center, University of Florida, Ona, FL 33865, USA; Range Cattle Research and Education Center, University of Florida, Ona, FL 33865, USA; Range Cattle Research and Education Center, University of Florida, Ona, FL 33865, USA; Range Cattle Research and Education Center, University of Florida, Ona, FL 33865, USA; Department of Agronomy, University of Wisconsin, Madison, WI, 53706, USA; Range Cattle Research and Education Center, University of Florida, Ona, FL 33865, USA; Range Cattle Research and Education Center, University of Florida, Ona, FL 33865, USA; Department of Animal Science, University of Florida, Gainesville, FL 32608, USA; Department of Animal Science, Para Federal University, Belem, PA 66075, Brazil; Range Cattle Research and Education Center, University of Florida, Ona, FL 33865, USA; Range Cattle Research and Education Center, University of Florida, Ona, FL 33865, USA

**Keywords:** warm-season perennial grass, rangelands, nutritive value, greenhouse gas

## Abstract

Prescribed fire is a common management practice used to manipulate rangeland plant productivity and composition. Although the nutritive value of most herbaceous plant species is considered poor for grazing animals, native rangelands in Florida are an important source of forage for livestock, especially during the winter months, when the productivity of cultivated perennial warm-season pastures is limited. This study evaluated the effects of prescribed fire on methanogenic potential and nutritive value of selected native rangeland plant species. Treatments were a 3 × 2 factorial arrangement of plant species (creeping bluestem [*Schizachyrium scoparium* var. stoloniferum {Nash} Wipff], wiregrass [*Aristida stricta* {Michx.}], or saw palmetto [*Serenoa repens* {W. Bartram} Small]) and prescribed fire management [2 yr after burning (control) vs. 1 yr after burning (burned)] distributed in a randomized complete block design with four replicates. Samples were analyzed for crude protein (CP), neutral detergent undigestible fiber (NDF), in vitro methane production, and in situ ruminal disappearance. Prescribed fire generally increased forage CP and DM effective degradability relative to control; however, no effect was observed on saw palmetto. Wiregrass had the least CP concentration in both burned (8.5%) and control (2.3%). In burned treatments, creeping bluestem and palmetto had greater DM effective degradability (62% and 58%) than wiregrass (53%). Fire increased in vitro gas production by 60 (creeping bluestem) to 90% (wiregrass) relative to control treatments. No effect of fire on methane production was observed for any of the plant species evaluated in this study. Creeping bluestem had the greatest methane production (12.5 mg/g DM), followed by wiregrass (5.3 mg/g DM) and saw palmetto (1.4 mg/g DM). Methane:DM effective degradability decreased in the following order: creeping bluestem ≥ wiregrass > saw palmetto. Data indicated prescribed fire was an effective tool to increase creeping bluestem and wiregrass nutritive value but no effect was observed on saw palmetto. Cattle grazing grass-dominated rangelands will likely emit more gas and methane than shrub or tree-dominated ecosystems; however, the greater forage nutritive value and subsequent positive impacts on animal production are expected to offset a substantial fraction of enteric methane emissions.

## Introduction

Native rangelands cover approximately 1.6 million ha in Florida and are important ecosystems for beef cattle production (Vendramini et al., 2006). This ecosystem also provides important economic, environmental, and cultural benefits to society. More than 300 species of plants exist in Florida flatwood rangelands; however, approximately 10 to 15 species make up most of the forage production (Vendramini et al., 2006). Creeping bluestem and wiregrass are the most predominant herbaceous species; while saw palmetto is the dominant shrub species. Although saw palmetto is not considered a forage species, the leaves represent a significant portion of the diet of cattle grazing Florida native rangelands (Kalmbacher et al., 1984). Differences in anatomy, morphology, and physiology affect chemical composition and digestibility of native forage species with subsequent impacts on livestock performance (Sollenberger et al., 2020). Moreover, differences in the quality of diets consumed by beef cattle can also have significant effect on enteric methane production and ecosystem greenhouse gas balance (Sanchez et al. 2022).

Prescribed fire is an important management tool to enhance and maintain rangeland ecosystem function (Archibald, 2008 and Thapa et al., 2022). Fire can also affect vegetation composition and nutritive value of native rangeland plant species (McIntosh et al. (2022); Raynor et al. 2021; Sanchez et al., 2022). Although several studies investigated the effects of prescribed fire on the nutritive value of rangelands (Archibald, 2008), limited information exists for predominant plant species in subtropical native rangeland ecosystems. Improving the use of low-quality forage by livestock can potentially increase animal performance with subsequent cobenefits in reducing enteric methane production. However, the mechanisms by which prescribed fire interactively alter the nutritive value and methanogenic potential of native forages species in Florida remains unknown.

Methane is a potent greenhouse gas with a global warming potential 28 to 29 times greater than carbon dioxide (Portner et al., 2022). Enteric methane emissions occur during ruminant digestion of fiber and are estimated to account for approximately 20% of the total methane anthropogenic emissions (US EPA, 2022). Globally, there is an increasing pressure for beef cattle production to reduce greenhouse gas emissions while also meeting increased demand for animal products. Native forage rangeland species often have relatively high NDF concentration and low digestibility compared with cultivated grass species, which may lead to greater enteric methane emissions (Congio et al., 2021). Therefore, management practices that improve the nutritional value of native rangeland plants may also result in reduction of methane emissions.

Although several studies investigated the impacts of pasture management on methanogenic potential of many forage species (Congio et al., 2021; Sanchez et al., 2022), there is limited information on fire management and its interactions with plant species in subtropical rangeland ecosystems. Therefore, the current study was designed to evaluate the effects prescribed fire on the nutritional value and methanogenic potential of selected Florida rangeland plant species.

## Material and Methods

The study was conducted at the University of Florida, Range Cattle Research and Education Center, Ona, Florida (27°26ʹN, 82°55ʹW), from August 2020 to February 2021. The predominant vegetation consisted of pine flatwood habitats, which represent the most extensive type of terrestrial ecosystem in Florida, covering approximately 50% of the natural land area (Vendramini et al. 2006). Predominant soils were Pomona (sandy, siliceous, hyperthermic Ultic Alaquod) and Ona (sandy, siliceous, hyperthermic Typic Alaquod) series. The experimental area has been subject to prescribed burning every 3 to 4 yr, with the last burning event occurring in February 2019. The site was also subjected to continuous stocking with nonlactating, nonpregnant beef cows (526 ± 67 kg body weight [BW]; 5.25 ha/cow) from December 2019 to March 2020 (∼90 d). Animal procedures used in this study were approved by the University of Florida Animal Care and Use Committee (#202111434). During the experimental period, mean temperature, rainfall, and solar irradiation were 27 °C and 25 °C, 246 and 71 mm, and 204 and 154 w/m^2^, for summer and autumn, respectively.

Treatments were a 3 × 2 factorial arrangement of plant species (creeping bluestem [*Schizachyrium scoparium* var. stoloniferum {Nash} Wipff], wiregrass [*Aristida stricta* {Michx.}] and saw palmetto [*Serenoa repens* {W. Bartram} Small]) and fire management (burned and control), distributed in a randomized, complete block design with four replicates. Control sites were burned in March 2019 (~2yr before sampling) while burned treatments were subjected to prescribed fire in March 2021 (~60 d before sampling). Plant species selection was based on previous studies that evaluated the contribution of each species to cattle diets (Kalmbacher, 1983; Kalmbacher et al., 1984). The experimental unit areas ranged from 17 to 26 ha. In each experimental unit, areas with the species of interest were marked with flags in May 2020 and sampled in May and June 2021. Caution was exercised to avoid resampling the same plants, previously marked with flags, which could affect nutritive value (Long et al. 1986).

Undisturbed canopy height measurements were taken in 20 random locations per sampling for each species using a measuring stick. A subsample of harvested material of each species was collected for determination of morphological composition. Samples were separated into leaf (leaf blades only), stem (including stems, sheaths, and inflorescence), and senescent material. Samples were dried at 55 °C for 72 h and weighed. Proportion of morphological components are presented as proportion of total mass.

Creeping bluestem was clipped to a 15-cm stubble height as indicated by Kalmbacher et al. (1986). Literature addressing stubble height recommendations for wiregrass was not found so plants were harvested at 15 cm stubble height to mimic the portion of the canopy grazed by cattle. The top 2/3 of the saw palmetto leaves were sampled based on Kalmbacher et al. (1984), who observed that cattle consumed the tips of saw palmetto fronds. Visual observations of recently grazed saw palmetto plants confirmed this report and indicated animals prefer upper leaves, usually harvesting around two-thirds of the leaflet length (distal part, severed at the point where leaflets separate from each other). Therefore, the first five expanded fronds of saw palmetto were clipped at the point where leaflets became separated. Because of the sampling protocol used for saw palmetto, no morphological composition assessment was performed for this plant species.

### Laboratory Analysis

Oven-dried samples were ground in a Wiley mill (Model 4, Thomas-Wiley Laboratory Mill, Thomas Scientific, Swedesboro, New Jersey) to pass a 1-mm stainless steel screen. Nitrogen concentration was determined by dry combustion using a LECO FP-528 Nitrogen Analyzer (LECO, St. Joseph, MI). crude protein (CP) was determined by multiplying N concentration by 6.25. The NDF concentration was analyzed according to ANKOM (2017b), with the inclusion of heat-stable α-amylase and sodium sulfite. Lignin concentration was determined by digesting samples in 72% sulfuric acid (ANKOM Technology 2020) after samples were analyzed for acid detergent fiber (ADF) in an ANKOM fiber analyzer (ADF) using the method of Van Soest et al. (1991) adapted for an ANKOM 200 Fiber Analyzer (ANKOM Technology, Macedon, NY)..

### In Situ Disappearance Kinetics

Samples were ground in a Wiley mill (Model 4, Thomas-Wiley Laboratory Mill, Thomas Scientific) to pass a 4-mm stainless steel screen. Four grams of ground material were placed in 20 × 10 cm, N-free nylon bags (10 mg/cm^2^ of bag surface) with pore sizes ranging from 50 to 60 μm. Two bags per experimental unit per incubation time (duplicates) were sealed with rubber bands and incubated for 0, 3, 6, 9, 12, 24, 48, and 96 h in two rumen-fistulated Braford steers (∼950 kg BW), each considered to be an incubation replicate. Steers were housed in individual pens through a 10-d adaptation period, followed by seven incubation periods, varying from 3 to 96 h. Animals had ad libitum access to chopped limpograss (*Hemarthria altissima* [Poir.] Stapf & C.E.Hubb) hay [8.5% CP, 52% in vitro digestible dry matter (DM; ANKOM, 2017a)] in addition to a daily supplementation with 1.5 kg of soybean meal (44% CP and 77% TDN). Limpograss was selected as the base forage diet for the animals due to the relatively low nutritive value, which is similar to the plant species selected in this study. Bags from each incubation period representing all the experimental units in the field were placed and removed from the steers simultaneously. After removal, bags were rinsed gently with de-ionized water until rinse was clear and stored at −20 °C until all incubation periods were completed. Initial bags (time zero/no incubation) were rinsed and stored similarly to incubated samples. At the end of the experiment, bags were thawed at room temperature and washed together in a typical washing machine used for clothes for one cycle of 25 min. (Foster et al., 2011). Lastly, bags were dried at 55 °C for 48 h and weighed. Dried samples were analyzed for total N and NDF concentration with the same procedures described for the nutritive value analyses.

The nonlinear model of Ørskov and McDonald (1979) was used to estimate CP, NDF, and DM disappearance kinetic parameters, as indicated in equation (1) below:


D = a + b 1 −e −kd (t) (1)

where *D* is the degradation at a time t (g/kg); *a* is the soluble fraction (g/kg); *b* is the potentially degradable fraction (g/kg); *kd* is the fractional rate of degradation of *b*; *t* is the incubation time (h). The variables *a*, *b*, and *kd* were estimated by nonlinear regression procedures of SAS (SAS 2013). The parameters used in the models are presented as descriptive data on Table 1.

**Table 1.** Dry matter (DM), crude protein (CP), and neutral detergent fiber (NDF) disappearance kinetic parameters for the nonlinear model described by Ørskov and McDonald (1979)

Response variable/Fire management	Species		
	Creeping bluestem	Wiregrass	Saw palmetto
DM			
	*a*		
Burned	38	39	47
Control	39	37	47
	*b*		
Burned	70	56	15
Control	60	27	32
	kd		
Burned	0.03	0.02	0.04
Control	0.02	0.10	0.03
CP			
	*a*		
Burned	40	47	38
Control	39	44	42
	*b*		
Burned	70	51	17
Control	64	30	49
	kd		
Burned	0.02	0.02	0.07
Control	0.01	0.04	0.04
NDF			
	*a*		
Burned	41	31	40
Control	33	33	38
		*b*	
Burned	69	63	17
Control	65	33	32
		kd	
Burned	0.02	0.03	0.06
Control	0.02	0.02	0.03

The fractionation method of Krishnamoorthy et al. (1983) was used to quantify CP, NDF, and DM in fractions A, B, and C. Fraction A represented the soluble and rapidly degraded fraction, and it was measured by setting the model to 0 h incubation. This fraction was assumed to be completely degraded. Fraction C was represented by the ruminally unavailable and undegraded material, and it was determined by setting the model to 96 h incubation. Fraction B, represented by the potentially degradable fraction, was measured by difference B = [100 – (A + C)]. Effective degradability was calculated using the model of Ørskov and McDonald (1979):


ED = a + b k d/ k d + k p (2)

where *a* is the fraction removed in the washing (%); *b* is the potentially degradable fraction (%); *kd* is the fractional rate of degradation of *b*; and *kp* is the fractional rate of passage of *b*. To calculate *ED* from the fractions evaluated in this experiment, *kp* (passage rate) was fixed as 0.02/h (Wallau et al. 2020).

### In Vitro Gas and Methane Production

*In vitro* gas production was measured using an ANKOM RF Gas Production System (ANKOM, Macedon, NY) equipped with nine modules. Each module consisted of a 250-mL bottle and an automated pressure/temperature sensor that recorded cumulative pressure every minute. Samples were incubated in four separate runs, where each run consisted of the samples of one experimental block (three plant species and two fire management) and a blank bottle. Rumen fluid was collected from a rumen-fistulated steer (∼950 kg BW) fed ad libitum stargrass (*Cynodon nlemfuensis* Vanderyst) hay and a daily supplementation of 1.8 kg of soybean meal (as-fed basis). The stargrass hay had similar nutritive value to the hay used in the in situ trial (9.3% CP and 53% in vitro digestible DM) but the steers were supplemented with slightly greater levels of soybean meal (1.8 vs. 1.5 kg/d); therefore, inferences in digestibility were made between treatments within the procedure. The fluid was collected from representative parts of the dorsal sack of the rumen, filtered through four cheesecloth layers, and immediately transported to the laboratory in a prewarmed, sealed container. A 4:1 McDougall’s buffer (McDougall 1948) rumen fluid solution was used as inoculum, added at the rate of 100 mL per bottle. Samples were ground to pass a 2-mm stainless steel screen and subsequently placed in each module at the rate of 2 g/bottle. In addition, 0.25 g of soybean (*Glycine max* L.) meal was added to each bottle to provide sufficient N for microbial activity during the fermentation process. Blank bottles received 0.25 g of soybean meal and the inoculum. Inoculated modules were maintained at 39 °C in a forced-air drier for 48 h.

Gas samples were collected with 1-L Tedlar bags (Supleco Analytical, Bellefonte, PA) connected to the modules. Samples were subsequently analyzed for methane concentration using an Agilent 7890 B gas chromatograph (Agilent Technologies Inc., Santa Clara, CA) equipped with autosampler and a flame ionization detector for methane determination. The total gas production and methane were expressed in term of either DM or DM effective degradability, estimated from the in situ procedure.

### Statistical Analysis

Response variables were analyzed using the MIXED procedure of SAS (SAS 2013). The model for nutritive value and gas production included plant species and fire management and their interaction as fixed effects and block and its interactions as random effects. The model for in situ disappearance response variables included plant species, fire management, and their interaction as fixed effects, whereas random effects included steer and block. Normality of residues and homogeneity of variances were tested using conditional studentized residual plots, and data were transformed if analysis of variance assumptions were violated. Means reported are nontransformed least square means. Treatments were considered different when *P* ≤ 0.05 by least significant difference test. Main effects and interactions not presented and discussed were not significant (*P* > 0.05).

## Results

### Canopy Height and Morphological Composition

There was a plant species and fire management effect on canopy height. Saw palmetto had the greatest canopy height but no differences were observed between creeping bluestem and wiregrass. Averaged across all species, prescribed fire decreased canopy height by approximately 30% relative to control (Table 2).

**Table 2.** Plant species and fire management effects on canopy height and morphological composition of native plants

Response variable	Fire management	Species			*Mean*	SE	*P*-value		
		Creeping bluestem	Wiregrass	Saw palmetto			Species	Fire	Species × Fire
Canopy height (cm)	Burned	28	26	67	40B^1^	6.3	<0.001	<0.001	0.085
	Control	42	35	97	58A				
	Mean	35b	30b	82a					
Leaf (% DM^2^)	Burned	96Aa	90Aa	na^3^	95	6.2	0.18	<0.001	0.03
	Control	52Ba	38Bb	na	63				
	Mean	74	64						
Stem (% DM)	Burned	1Ab	5Aa	na	3	0.6	0.03	0.12	0.03
	Control	1Aa	1Ba	na	1				
	Mean	1	3						
Senescent material (% DM)	Burned	3Ba	5Ba	na	4	6.2	0.48	<0.001	<0.001
	Control	47Ab	61Aa	na	54				
	Mean	25	33						

^1^Means within columns followed by the same uppercase letter are not different (*P* > 0.05). Means within rows followed by the same lowercase letter are not different (*P* > 0.05).

^2^DM, dry matter.

^3^na, not applicable. Because of the sampling protocol used for saw palmetto, no morphological composition assessment was performed for this plant species.

There was a plant species × fire management interaction effect on leaf, stem, and senescent material proportions (Table 2). There was no difference in leaf proportion between creeping bluestem and wiregrass in the burned treatment; however, in the control treatments, creeping bluestem had the greatest leaf proportion. Prescribed fire generally increased leaf proportion and decreased senescent material of both plant species relative to control treatments. In the burned treatments, creeping bluestem had less stem proportion than wiregrass; however, there was no difference between wiregrass and creeping bluestem in the control treatments. Fire increased wiregrass stem proportion relative to control treatment but no effect was observed for creeping bluestem.

Wiregrass had greater senescent material proportion than creeping bluestem in control treatment; however, no difference was observed in burned treatments.

### Nutritive Value

CP concentration was affected by plant species × fire management interaction (Table 3). Prescribed fire increased creeping bluestem and wiregrass CP concentrations by 2.2- and 3.7-fold, respectively, but no effect of fire on CP was observed for saw palmetto. Saw palmetto had the greatest CP concentration in the control treatment; however, in burned treatments no difference in CP concentration between saw palmetto and creeping bluestem was observed. In both burned and control treatments, wiregrass had the lowest CP concentrations among the plant species.

**Table 3.** Plant species and fire management effects on crude protein (CP), neutral detergent fiber (NDF), acid detergent fiber (ADF), and lignin concentration of native plants

Response variable	Fire management	Species			Mean	SE	*P*-value		
		Creeping bluestem	Wiregrass	Saw palmetto			Species	Fire	Species × Fire
CP (% DM^1^)	Burned	9.8Aa^2^	8.5Ab	10.6Aa	9.6	0.31	<0.01	<0.01	<0.01
	Control	4.4Bb	2.3Bc	7.7Aa	4.8				
	Mean	7.1	5.4	9.1					
NDF (% DM)	Burned	81	73	84	79	0.6	<0.01	0.43	0.07
	Control	80	69	86	78				
	Mean	80b	71c	84a					
ADF (% DM)	Burned	38	42	39	39	0.6	<0.01	0.11	0.06
	Control	44	46	38	42				
	Mean	41b	39c	44a					
Lignin (% DM)	Burned	2.1	4.4	11.2	5.9	0.4	<0.01	0.11	0.11
	Control	4.4	6.2	11.3	7.3				
	Mean	3.8c	5.3b	11.3a					

^1^DM, dry matter.

^2^Means within columns followed by the same uppercase letter are not different (*P* > 0.05). Means within rows followed by the same lowercase letter are not different (*P* > 0.05).

There was a species effect on NDF, ADF, and lignin concentrations. Saw palmetto had the greatest NDF and ADF concentrations, followed by creeping bluestem, and wiregrass. Saw palmetto had the greatest lignin concentration, followed by wiregrass, and creeping bluestem had the least lignin concentration.

### In Situ Ruminal Disappearance

There was no treatment effect on DM fraction A; however, plant species affected DM fraction B. The DM fraction B did not differ between creeping bluestem and wiregrass but both species were greater than saw palmetto.

There was a plant species × fire management interaction effects for DM Fraction C and effective degradability (Table 4). Although no plant species effects were observed in control treatments, saw palmetto had greater DM fraction C than creeping bluestem in the burned treatment. Prescribed fire decreased wiregrass DM fraction C, but no effect of fire was observed for the other species. In the burned treatment, there was no difference in DM effective degradability between creeping bluestem and saw palmetto, which were greater than wiregrass. However, in the control treatment, creeping bluestem had the greatest DM effective degradability and there was no difference between wiregrass and saw palmetto. Fire increased creeping bluestem and wiregrass DM effective degradability by 21% to 29%, respectively but no effect was observed for saw palmetto.

**Table 4.** Plant species and fire management effects on dry matter (DM) fractions A (readily degradable in the rumen), B (potentially degradable in the rumen), and C (undegradable in the rumen) and effective degradability of native plants

Response variable	Fire management	Species			Mean	SE	*P*-value		
		Creeping bluestem	Wiregrass	Saw palmetto			Species	Fire	Species × Fire
Fraction A (% DM)	Burned	28	22	27	26	2,0	0.09	0.13	0.33
	Control	19	18	27	21				
	Mean	23	20	27					
Fraction B (% DM)	Burned	40	40	15	31	2.2	0.03	0.09	0.06
	Control	33	24	18	25				
	Mean	36a^1^	32a	16b					
Fraction C (% DM)	Burned	48Ab	41Bab	58Aa	49	0.3	0.11	0.22	0.03
	Control	48Aa	58Aa	54Aa	53				
	Mean	48	49	56					
Effective degradability (% DM)	Burned	62Aa	53Ab	58Aa	5.9	1.2	<0.01	<0.01	0.02
	Control	51Ba	41Bb	45Ab	7.3				
	Mean	56	47	45					

^1^Means within columns followed by the same uppercase letter are not different (*P* > 0.05). Means within rows followed by the same lowercase letter are not different (*P* > 0.05).

There was a plant species effect on CP Fraction A (Table 5). Wiregrass had the greatest CP Fraction A, while creeping bluestem and saw palmetto had similar but lesser CP fraction A than wiregrass. There was no effect of plant species and fire management on CP fraction B (Table 5). However, there was a plant species × fire management interaction effect on CP Fraction C (Table 4). Although CP fraction C was similar among species in the control treatment, in the burned treatment saw palmetto had the greatest CP fraction C than wiregrass and creeping bluestem. Prescribed fire reduced wiregrass and creeping bluestem CP fraction C while it increased CP fraction in saw palmetto. Wiregrass and creeping bluestem had greater CP effective degradability than saw palmetto.

**Table 5.** Plant species and fire management effects on crude protein (CP) fractions A (readily degradable in the rumen), B (potentially degradable in the rumen) and C (undegradable in the rumen) and effective degradability of native plants

Response variable	Fire management	Species			Mean	SE	*P*-value		
		Creeping bluestem	Wiregrass	Saw palmetto			Species	Fire	Species × Fire
Fraction A (% CP)	Burned	20	27	18	21	1.5	0.02	0.58	0.11
	Control	20	21	19	20				
	Mean	20b^1^	24a	19b					
Fraction B (% CP)	Burned	46	35	27	32	5.0	0.14	0.26	0.10
	Control	29	19	28	25				
	Mean	37	27	22					
Fraction C (% CP)	Burned	33Bb	38Bb	65Aa	45	4.0	0.02	0.07	0.03
	Control	50Aa	59Aa	55Ba	55				
	Mean	41	48	60					
Effective degradability (% CP)	Burned	56	56	40	50	2.3	<0.01	0.08	0.07
	Control	45	53	41	46				
	Mean	51a	54a	40b					

^1^Means within columns followed by the same uppercase letter are not different (*P* > 0.05). Means within rows followed by the same lowercase letter are not different (*P* > 0.05).

Plant species and fire management did not affect NDF Fraction A; however, there was a plant species effect for NDF fractions B and C (Table 6). The NDF Fraction B of creeping bluestem and wiregrass did not differ but was greater than saw palmetto. Conversely, saw palmetto had the greatest NDF fraction C among the species. There was a plant species and fire management effect of NDF effective degradability (Table 6). Fire increased NDF effective degradability. Creeping bluestem had the greatest NDF effective degradability followed by saw palmetto and wiregrass.

**Table 6.** Plant species and fire management effects on neutral detergent fiber (NDF) fractions A (readily degradable in the rumen), B (potentially degradable in the rumen), and C (undegradable in the rumen) and effective degradability of native plants.

Response variable	Fire management	Species			Mean	SE	*P*-value		
		Creeping bluestem	Wiregrass	Saw palmetto			Species	Fire	Species × Fire
Fraction A (% NDF)	Burned	21	11	20	18	3.0	0.10	0.31	0.25
	Control	13	13	18	15				
	Mean	17	19	13					
Fraction B (% NDF)	Burned	25	40	17	27	3.6	<0.01	0.45	0.06
	Control	33	26	16	25				
	Mean	29a^1^	33a	16b					
Fraction C (% NDF)	Burned	57	48	62	55	2.0	0.02	0.18	0.12
	Control	54	60	64	59				
	*Mean*	56b	54b	64a					
Effective degradability (% NDF)	Burned	58	40	50	49A	2.0	<0.01	<0.01	0.17
	Control	48	30	36	39B				
	Mean	53a	43b	38c					

^1^Means within columns followed by the same uppercase letter are not different (*P* > 0.05). Means within rows followed by the same lowercase letter are not different (*P* > 0.05).

### In Vitro Gas and Methane Production

There was a plant species × fire management interaction effect on gas production (Table 7). Prescribed fire generally increased in vitro gas production (60% and 100% increase for creeping bluestem and wiregrass, respectively). No effect of fire on gas production was observed for saw palmetto. Creeping bluestem had the greatest gas production, followed by wiregrass, and saw palmetto had the least gas production in both burned and control treatments.

**Table 7.** Plant species and fire management effects on in vitro gas and methane production of native plants

Response variable	Fire management	Species			Mean	SE	*P*-value		
		Creeping bluestem	Wiregrass	Saw palmetto			Species	Fire	Species × Fire
Gas production (mL/g DM^1^)	Burned	175Aa^2^	116Ab	42Ac	111	5.0	<0.01	0.01	<0.01
	Control	110Ba	58Bb	34Ac	67				
	Mean	142	86	38					
Methane (mg/g DM)	Burned	16.3	6.9	2.1	8.4	1.4	0.01	0.37	0.40
	Control	8.7	3.7	0.8	4.4				
	Mean	12.5a	5.2b	1.4c					
Methane:gas production ratio	Burned	0.09	0.06	0.05	0.07	0.02	0.01	0.37	0.40
	Control	0.07	0.06	0.02	0.05				
	Mean	0.08a	0.06ab	0.03b					
Gas production:DM effective degradability	Burned	307Aa	230Ab	106Ac	214	9.0	<0.01	0.01	0.01
	Control	227Ba	161Bb	97Ac	161				
	Mean	267	195	101					
Methane:DM effective degradability	Burned	17	11	12	13	2.5	0.04	0.99	0.07
	Control	16	17	6	13				
	Mean	17a	14ab	9b					

^1^DM, dry matter.

^2^Means within columns followed by the same uppercase letter are not different (*P* > 0.05). Means within rows followed by the same lowercase letter are not different (*P* > 0.05).

There was a plant species effect on methane and methane:gas production ratio (Table 7). Creeping bluestem had the greatest methane production, followed by wiregrass and saw palmetto. Similar proportion of methane:gas production was observed for creeping bluestem. (Table 7). Fire increased gas production:DM effective degradability for creeping bluestem and wiregrass but no effect was observed for saw palmetto. The greatest gas production:DM effective degradability was associated with creeping bluestem followed by wiregrass and saw palmetto. Methane:DM effective degradability decreased in the following order: creeping bluestem ≥ wiregrass > saw palmetto.

## Discussion

### Canopy Height and Morphological Composition

Regardless of the plant species evaluated herein, the effect of fire in reducing canopy height persisted for 60 d after the prescribed fire was imposed. Since both creeping bluestem and wiregrass are warm-season perennial bunch grasses from the same family (Poaceae), these species exhibited similar morphology. Saw palmetto, however, consisted of a shrub species from the Arecaceae family and is characterized by different canopy structure and morphology than the grasses evaluated in this study. The canopy composition of creeping bluestem and wiregrass in the burned treatment was primarily leaves, while in control treatment, senescent material represented a significant proportion of DM in both species. Fire reduced senescent material and increased leaf proportion but negligible effect was observed on stem proportion.

Greater creeping bluestem and wiregrass CP concentration in burned treatments is likely due to the combustion and removal of the senescent material, which has decreased CP concentration (Akin, 1989). In addition, fire increases soil N availability which can also increase plant CP concentration (Kohmann et al., 2022). Increases in CP concentration after prescribed fire has also been reported in several studies (Archibald and Bond, 2004; Anderson et al., 2007; Thapa et al., 2021). Lack of fire effect of saw palmetto CP concentrations was likely due the relatively high CP concentrations relative to creeping bluestem and wiregrass. This response was also consistent with results that were observed by Sanchez et al. (2022). Since only saw palmetto leaflets were evaluated in the current study, it is also plausible that the effect of fire on other plant parts was masked (Pitman, 1993). The initial CP concentration in all species evaluated in this study were sufficient to meet the CP requirements of nonlactating mature beef cows (NASEM 2016).

Fire management did not affect NDF, ADF, and lignin concentrations, indicating that the dead senescent material likely had similar hemicellulose, cellulose, and lignin concentration of the leaves and stems. However, there were differences among plant species. Creeping bluestem had greater NDF, but lesser ADF, indicating that a greater proportion of the fiber was hemicellulose, which is a more digestible portion of the fiber (Van Soest, 1994). Greater NDF concentration observed in saw palmetto was due to greater lignin concentration, which is expected to reduce fiber and overall forage digestibility (Van Soest, 1994). Saw palmetto NDF and ADF concentrations observed herein are similar to those previously reported in other studies in Florida (Kalmbacher, 1983; Pitman 1993; Sanchez et al. 2022).

The effect of prescribed fire on creeping bluestem and wiregrass DM effective degradability was likely due to the increase in leaf blade proportions, which typically contain greater concentration of soluble components (Alencar et al., 2019). In addition, fire decreased the senescent material, which usually have lesser cell contents (Akin, 1989). Although there was no effect of fire on creeping bluestem DM fraction A and B, the means were approaching difference and when combined in the DM effective degradability calculation, creeping bluestem in burned treatments had greater DM effective digestibility than control. Creeping bluestem had greater hemicellulose and lesser lignin concentration than wiregrass and saw palmetto, resulting in greater DM effective degradability. Corroborating with this study, Sanchez et al. (2022) observed that creeping bluestem had the greatest DM effective degradability among native forage species. According to these authors, this result was attributed to the greatest DM Fraction B and least Fraction C. Greater concentration of DM Fraction B in creeping bluestem is likely due to greater concentrations of starch, sugar, and pectin (Hastert et al., 1983). Greater effective degradability in wiregrass in burned treatments was likely due to the increases in DM fraction B and decrease in fraction C. Saw palmetto had lesser DM effective degradability than creeping bluestem, due to greater lignin concentration and proportion of fraction C. The effective degradability creeping bluestem and wiregrass in burned treatments is above the requirement of a nonlactating mature beef cow (520 g\kg; NASEM 2006).

The CP fractions are affected by several abiotic and biotic factors, such as species, maturity, season, fertilization, and biological N fixation (Vendramini et al., 2008; Sanchez et al., 2018). In warm-season perennial grasses, there is a decrease in CP fractions A and B and increase in CP fraction C due to increase in NDF, ADF, and lignin concentrations, which liked led to greater amount of CP linked to the fiber and not degraded in the rumen (Vendramini et al., 2008). Although prescribed fire generally increased CP concentration relative to control treatments, it is plausible that the lack of the fire management effect on CP fractions and degradability may be due to the lack of fire effect on ADF and NDF concentrations (Patel and Berr, 2008). Saw palmetto exhibited the least CP fractions A, C, and effective degradability and the greatest NDF and lignin concentrations. These data corroborate with previous studies in Florida rangelands which indicated that saw palmetto has greater lignin and fiber concentrations than native grasses, such as wiregrass and creeping bluestem (Kalmbacher et al. 1984; Pitman 1993; Sanchez et al. 2022). Differences in CP fractions and effective digestibility among species observed in the current study are of great importance to design adequate CP supplementation programs for ruminants grazing native rangelands in Florida.

Although there was no effect of prescribed fire on NDF A, B, and C fractions, when NDF fractions A and B are used in the effective degradability calculations, it resulted in significant increase in NDF effective degradability with fire for all species evaluated in this study. Nonetheless, wiregrass had lesser NDF fraction B and greater fraction C than creeping bluestem, resulting in lesser NDF effective degradability. Differences in NDF fractions B and C between creeping bluestem and wiregrass are likely due to differences in cell wall composition. Creeping bluestem had greater proportion of hemicellulose and lesser proportion of lignin in the cell wall than wiregrass. Saw palmetto had greater NDF concentration than wiregrass and creeping bluestem; however, the NDF effective digestibility was intermediate to creeping bluestem and wiregrass, and comparable to several warm-season perennial grasses (Vendramini et al. 2010). Fire increased NDF effective degradability in creeping bluestem, wiregrass, and saw palmetto. This was likely due to morphological changes that affected the proportion and composition of NDF (Vendramini et al., 2010; Delevatti et al., 2019). The NDF fractions observed in this study are consistent with previous reports in rangelands (Allred et al., 2011; Sanchez et al., 2022; Thapa et al., 2022). The greatest NDF Fraction B in saw palmetto did not result in increased NDF effective degradability. It is likely that the greatest lignin concentration in saw palmetto negatively affected NDF effective degradability. The NDF concentration and digestibility can be highly variable depending on the plant species and are poor predictors of DM digestibility (da Silva et al., 2020).

### In Vitro Gas and Methane Production

Greater gas production in creeping bluestem and wiregrass than saw palmetto in both burned and control treatments is due to differences in DM effective degradability among these species. Forage species with greater digestibility are often associated with greater rumen-microbial activity and gas production (du Toit et al., 2018; Berça et al., 2019). However, prescribed fire increased gas production in creeping bluestem and wiregrass but did not affect saw palmetto gas production. Fire increased gas production:DM effective degradability in creeping bluestem and wiregrass; however, it did not affect saw palmetto due to the lack of fire effect on saw palmetto gas production. This may be due to the presence of recalcitrant compounds in saw palmetto leaves, such as wax and tannic acid (Kalmbacher et al. 1984; Bennett and Hicklin, 1998) that are typically not fermented by rumen microbes. According to Boufennara et al. (2019), ruminal fermentation decreases in the presence of secondary metabolism compounds such as condensed tannins in palm species. The greater gas production:DM effective degradability ratio in creeping bluestem than wiregrass and saw palmetto indicated that the increase gas production in creeping bluestem was disproportionally greater than the increase in DM effective digestibility.

Fiber fermentation is the major contributor of enteric methane production. Sekine et al. (1986) observed that hay with greater nutritive value resulted in greater methane production due to greater digestible fiber. In addition, Moe and Tyrrell (1979) observed that the amount of digested cellulose contributes to methane production more than other carbohydrate components. Although fire had no effect on NDF concentration and methane production, prescribed fire increased NDF effective degradability. This response implies that the magnitude of increase in NDF effective degradability was likely not sufficient to increase methane production. However, plant species with greater NDF concentration and NDF effective digestibility, such as creeping bluestem and wiregrass had greater methane production. Similarly, creeping bluestem had greater methane:DM effective degradability ratio than saw palmetto. The proportion of methane:gas production is often used as an indicator of grass fermentability efficiency (du Toit et al., 2018). Methane:gas production values reported in the current study were similar to those reported for subtropical grass species (du Toit et al., 2018; Sanchez et al., 2022).

## Conclusion

Prescribed fire was an effective management practice to increase overall nutritive value of native forage species in Florida rangelands. Results demonstrated that there were significant differences in morphology and nutritive value among species and accurate estimate of botanical and chemical composition of rangeland plant species is key to optimize animal performance.

CP supplementation is an important management practice for ruminants grazing rangelands. Although CP concentration of most native plant species was within the sufficient level to meet livestock requirements, limited CP effective digestibility should be taken into consideration when designing supplementation program for ruminants grazing native plant species.

Native grasses generally had greater DM and CP effective degradability than palmetto. This result implies that different supplementation strategies may be required to meet the nutritional requirements of cattle grazing rangelands dominated by different vegetation types.

Although saw palmetto resulted in less gas and methane production than native grass species, the relatively poor nutritive value is expected to limit the proportion of saw palmetto in the livestock diet. Cattle grazing grass-dominated rangelands may have greater gas and methane production than shrub or tree-dominated ecosystems; however, the greater forage nutritive value and subsequent positive impacts on animal production are expected to offset a substantial fraction of enteric methane emissions.
